# Gray wolf optimizer with bubble-net predation for modeling fluidized catalytic cracking unit main fractionator

**DOI:** 10.1038/s41598-022-10496-2

**Published:** 2022-05-09

**Authors:** Xiaojing Wang, Chengli Su, Ning Wang, Huiyuan Shi

**Affiliations:** 1 School of Information and Control Engineering, Liaoning Petrochemical University, Fushun, 113000 PR China; 2 State Key Laboratory of Industrial Control Technology, Zhejiang University, Hangzhou, 310027 PR China

**Keywords:** Chemical engineering, Computer science

## Abstract

Fluidized catalytic cracking unit (FCCU) main fractionator is a complex system with multivariable, nonlinear and uncertainty. Its modeling is a hard nut to crack. Ordinary modeling methods are difficult to estimate its dynamic characteristics accurately. In this work, the gray wolf optimizer with bubble-net predation (GWO_BP) is proposed for solving this complex optimization problem. GWO_BP can effectively balance the detectability and exploitability to find the optimal value faster, and improve the accuracy. The head wolf has the best fitness value in GWO. GWO_BP uses the spiral bubble predation method of whale to replace the surrounding hunting scheme of the head wolf, which enhances the global search ability and speeds up the convergence speed. And Lévy flight is applied to improve the wolf search strategy to update the positions of wolfpack for overcoming the disadvantage of easily falling into local optimum. The experiments of the basic GWO, the particle swarm optimization (PSO) and the GWO_BP are carried out with 12 typical test functions. The experimental results show that GWO_BP has the best optimization accuracy. Then, the GWO_BP is used to solve the parameter estimation problem of FCCU main fractionator model. The simulation results show that the FCCU main fractionator model established by the proposed modeling method can accurately reflect the dynamic characteristics of the real world.

## Introduction

Fluid catalytic cracking is one important part of the petroleum refining processes. The heavy oil is reacted in a lifting tube having a temperature of about 500 °C in the presence of a molecular sieve catalyst. The hydrocarbons of the born can be separated into cracking gas, high octane gasoline and diesel. It is one of the important measures to convert heavy oil into light oil. The catalytic cracking main fractionation column is a master device that achieves product separation during catalytic cracking, which is an important part of catalytic cracking. Therefore, establishing a precise main fractionator model has a great significance to improve oil product quality^1^. The catalytic crack process is full of uncertainty, complexity, nonlinearity and coupling among variables etc.^2,3^. It is very difficult to obtain an accurate mathematical model by traditional modeling methods, they have been unable to meet its requirements of building a high-precision mathematical model^4,5^. The modeling problem of complex system can be transformed into a complex optimization problem. Researchers found that intelligent optimization algorithms can solve the complex optimization problems^6–8^. Intelligent optimization algorithms have no strict requirements for optimization problems, and easy to operate and implement^9^.

With the development of artificial intelligence technique, intelligent optimization algorithms are also used in petroleum industry modeling^10^. Li et al. applied Tabu Search to solve chemical industry optimization problems, and obtains the global optimal solution in specific constrained optimization problems by setting different parameters^11^. Tao and Wang proposed an RNA genetic algorithm to estimate the parameters of a heavy oil thermal cracking model and a fluid catalytic cracking unit (FCCU) main fractionator^12^. Li et al. used grey wolf optimizer (GWO) to optimize multi-classification Twin Support Vector Machine to explore concealed reservoirs^13^. Hu et al. used a normal distribution function to improve the search mode of fruit fly optimization algorithm to predict the oil pipeline energy consumption^14^.

Inspired by the preying activity of gray wolf, grey wolf optimizer (GWO) is proposed in 2014^15^. GWO has strong search performance, few parameters and easy implementation. Some researchers used GWO to optimize the model parameters, Altan et al. established the wind speed forecasting hybrid model and optimized the intrinsic mode function estimation output with the GWO^16^. However, the basic GWO still has some defects, such as premature convergence, easy to fall into local optimum and so on. In order to overcome the defects, researchers have put forward various GWOs. Malik et al. used the weighted average to update the position of wolfpack and improve the diversity of GWO population^17^. Yao et al. used the "greedy strategy" in differential evolution to eliminate the poorer new individual positions in the wolves for the UAV three-dimensional path planning problem^18^. Heidari et al. integrated Lévy flight (LF) and greedy selection strategies to the modified hunting phases^19^. Amirsadri et al. used Lévy flight for the encirclement formula of α, β and δ wolves in GWO and combined BP algorithm to solve the global optimization problem^20^. Gupta et al. proposes a modified GWO by incorporating random walk for leading wolves to optimize the search ability for prey by wolf pack^21^. Wang et al. used differential evolution and elimination mechanisms to update the wolfpack for speeding up the convergence rate and improving the convergence accuracy^22^. Akash et al. performed an experiment of embedding β-Chaotic sequence in monotonically decreasing linear mechanism to avoid falling into local optimization^23^. Nadimi-Shahraki et al. used the dimension learning hunting search strategy to improve GWO, so that each wolf constructs a neighborhood, and the adjacent information is shared among wolves, which alleviated the lack of population diversity, the imbalance between development and exploration and the premature convergence^24^. Liu and Wang proposed a GWO with RNA cross operation to improve the global optimization ability and changed the adaptive parameter to balance the exploration and development ability^25^. The improved methods as above provide the reference values. The global search ability (detectability) and local search ability (exploitability) of GWO needs to study for reaching an ideal balance. The method proposed in this work can efficiently balance the detectability and exploitability. In this work, in order to overcome the shortcomings of basic GWO, we combine the whale search scheme and the Lévy flight for the head wolf $\alpha$ search strategy to propose the novel gray wolf optimizer (GWO_BP), and adopt GWO_BP to estimate the parameters of the FCCU main fractionator model. The highlights of this paper are as follows:

- The gray wolf optimization with bubble-net predation (GWO_BP) is proposed.
- In order to enhance the global search ability and accelerate the convergence speed, the bubble-net predation of whale search scheme is applied to update the head wolf $\alpha$ position for GWO_BP.
- And the Lévy flight is used to the head wolf $\alpha$ to update the positions of wolfpack for overcoming the disadvantage of easily falling into local optimum.
- GWO combined with whale bubble predation and Lévy flight for head wolf $\alpha$ search strategy is applied to the parameter estimation of FCCU main fractionator model. The experimental results show that the proposed modeling method can track the dynamic characteristics well.

The chapter structure is as follows. The second section introduces the basic GWO. The third section proposes a novel GWO (GWO_BP), which merge the whale bubble-net predation search scheme and Lévy flight. The fourth section uses the typical optimization test functions to test the GWO_BP and the basic GWO and PSO. The GWO_BP is applied to estimate the model parameter of FCCU main fractionator to verify the effectiveness and feasibility.

## Basic gray wolf optimizer

GWO is a newly intelligence optimization algorithm proposed by Mirjalili et al.^15^. GWO is inspired by the prey hunting activities of gray wolves. Gray wolves are social canine animals that live at the top of the food chain, and they adhere to a strict hierarchy of social dominance. There are four social hierarchies in the gray wolf population $\alpha$, $\beta$, $\delta$, $\omega$. Where $\alpha$ is the leading wolf in the wolf pack, and it makes major decisions about activities in the wolves, In the GWO, $\alpha$ is the best fitness value in the wolves. $\beta$ wolf obeys the leading wolf and helps him to make decisions. $\beta$ wolf can dominate the other grades of wolves, and $\beta$ is the second best solution of fitness value in GWO. $\delta$ obeys $\alpha$ and $\beta$ in the wolf pack, and at the same time dominates the wolves of the remaining level. $\delta$ is the third best solution of fitness value. The rest of the candidate solutions are $\omega$, $\omega$ wolves usually need to obey wolves at higher social levels. The optimization process of GWO is guided by $\alpha$, $\beta$ and $\delta$, after judging the position of the prey which is the optimal solution, $\alpha$, $\beta$ and $\delta$ lead $\omega$ to surround the prey. Finally, the optimal value is found by iterating continuously.

GWO can divide the whole process of hunting prey into three stages, which are encircling, hunting, attacking, and finally capturing the prey, The detailed algorithm is described as follows^15^:

1. Encircling prey: after the wolves lock the location of the prey, they will slowly move to the prey for encirclement. In the encirclement process, the distance between the gray wolf and the prey can be calculated by Eqs. (1) and (2)^15^:
  1 $$\overrightarrow {D} = \left| {\overrightarrow {C} \cdot \overrightarrow {X}_{P} (t) - \overrightarrow {X} (t)} \right|$$
  2 $$\overrightarrow {X} (t + 1) = \overrightarrow {X}_{P} (t) - \overrightarrow {A} \cdot \overrightarrow {D}$$

where $\overrightarrow {D}$ indicates the distance between the wolf and prey, $t$ is the current iteration, $\overrightarrow {X}_{P} (t)$ is the location of prey after the iteration $t$, $\overrightarrow {A}$ and $\overrightarrow {C}$ are coefficients, and can be calculated as follows^15^:

3 $$\overrightarrow {A} = 2\overrightarrow {a} \cdot \overrightarrow {r}_{1} - \overrightarrow {a}$$

4 $$\overrightarrow {C} = 2 \cdot \overrightarrow {r}_{2}$$

where $\overrightarrow {r}_{1}$, $\overrightarrow {r}_{2}$ are random value in [0,1]. $\overrightarrow {a}$ is the convergence factor and decreases linearly from 2 to 0 with the iteration proceeds, the definition is as follows^17^:

5 $$\overrightarrow {a} = 2 - \frac{2t}{{T_{\max } }}$$

(2) Hunting: the hunting process of gray wolf population is usually guided by the head wolf $\alpha$, $\beta$ and $\delta$ assisted the wolf $\alpha$. Therefore, GWO assumes that $\alpha$, $\beta$ and $\delta$ are related to the possible positions of prey, and updates according to the positions of these three optimal solutions, the expression are as follows^15^:
  6 $$\left\{ {\begin{array}{*{20}l} {\overrightarrow {D}_{\alpha } { = }\left| {\overrightarrow {C}_{1} \cdot \overrightarrow {X}_{\alpha } (t) - \overrightarrow {X} (t)} \right|} \hfill \\ {\overrightarrow {D}_{\beta } { = }\left| {\overrightarrow {C}_{2} \cdot \overrightarrow {X}_{\beta } (t) - \overrightarrow {X} (t)} \right|} \hfill \\ {\overrightarrow {D}_{\delta } { = }\left| {\overrightarrow {C}_{3} \cdot \overrightarrow {X}_{\delta } (t) - \overrightarrow {X} (t)} \right|} \hfill \\ \end{array} } \right.$$
  7 $$\left\{ {\begin{array}{*{20}l} {\overrightarrow {X}_{1} = \overrightarrow {X}_{\alpha } (t) - \overrightarrow {A}_{1} \overrightarrow {D}_{\alpha } } \hfill \\ {\overrightarrow {X}_{2} = \overrightarrow {X}_{\beta } (t) - \overrightarrow {A}_{2} \overrightarrow {D}_{\beta } } \hfill \\ {\overrightarrow {X}_{3} = \overrightarrow {X}_{\delta } (t) - \overrightarrow {A}_{3} \overrightarrow {D}_{\delta } } \hfill \\ \end{array} } \right.$$
  8 $$\overrightarrow {X} (t + 1) = \frac{{\overrightarrow {X}_{1} + \overrightarrow {X}_{2} + \overrightarrow {X}_{3} }}{3}$$
(3) Attack prey: This step is the last stage of the hunting process, the wolves siege and capture the prey (obtain the optimal solution). This stage can be realized by decreasing the value of $\overrightarrow {a}$ in Eq. (3). When the value of $\overrightarrow {a}$ decreases linearly from 2 to 0, the corresponding value of $\overrightarrow {a}$ also changes in the interval [− a, a]. When the random value of *A* is above [− 1,1], the next position of the wolf may be anywhere between its current position and its prey position, when |*A*|> 1, wolves are currently moving away from position of prey to find new potential prey. Random parameter *C* in Eq. (4) ranges from [0,2], parameter *C* randomly enhances (*C* ≥ 1) or weakens (*C* < 1) the influence of the target wolf on the computational distance, this helps to enhance the detectability of the algorithm and avoid local optimum values.

### Gray wolf optimizer with bubble-net predation

#### The bubble-net predation of whales

When the basic GWO encircles the prey, it will gradually make the wolves approach the $\alpha$, $\beta$, $\delta$ wolves through continuous iteration, which may reduce the population diversity and lead the algorithm falling into the local optimal values. It is unfavorable for solving the problems with multiple local optimal values. We turn our attention to the whale foraging behavior, and make use of the bubble-net predation of whales to enhance the leading wolf $\alpha$.

Whales are the largest mammals in the world, and they have a unique method of foraging, called the bubble feeding method. Whales dive into the ocean about 12 m, then create a spiral bubble around their prey, and then swim to the surface. Inspired by this foraging behavior, whale optimization algorithm (WOA) is proposed by Mirjalili et al.^26^. The advantages of WOA are simple operation, fewer parameters to adjust good global optimization ability and the convergence speed is fast. The bubble net predation is expressed by the following formula^26^:

9 $$\overrightarrow {X} (t + 1) = \overrightarrow {{X^{*} }} (t) + \overrightarrow {{D}_{p}} \cdot\ e^{bl} \cos (2\pi l)$$

where $\overrightarrow {{D_{p} }} = \left| {\overrightarrow {X}^{*} \left( t \right) - \overrightarrow {X} \left( t \right)} \right|$ represents the length of the *i*th whale from its prey, that is, the distance between the *i*th solution and the current optimal solution; $\overrightarrow {X}^{*} (t)$ represents the best whale position up to now, $\overrightarrow {X} (t)$ represents the current position of the whale; $b$ is a constant, usually $b$ takes 1, which determines the shape of the diameter spiral; $l$ is the random value on [− 1, 1], and ‘∙’ represents the dot product.

In addition, the algorithm believes that whales also surround their prey, which is similar to the encirclement mechanism of GWO, the mathematical description is as follows^26^:

10 $$\overrightarrow {X} (t + 1) = \overrightarrow {X}^{*} (t) - \overrightarrow {A} \cdot \overrightarrow {D}$$

Suppose we update the whale's position with probabilistic selective contraction encirclement mechanism of $P_{i}$ and probability selection spiral model of Eq. (9). WOA sets when $A < 1$, the whale attacked its prey, the mathematical formula is as follows^26^:

11 $$\overrightarrow {X} (t + 1) = \left\{ {\begin{array}{*{20}l} {\overrightarrow {X}^{*} (t) - \overrightarrow {A} \cdot \overrightarrow {D} ,} \hfill & {p < Pi} \hfill \\ {\overrightarrow {X} (t) = \overrightarrow {X}^{*} (t) + \overrightarrow {{D}_{p}}\cdot \ e^{bl} \cos (2\pi l),} \hfill & {p \ge Pi} \hfill \\ \end{array} } \right.$$

where, $\overrightarrow {D} = \left| {\overrightarrow {C} \cdot \overrightarrow {X}^{*} \left( t \right) - \overrightarrow {X} \left( t \right)} \right|$ is the radius of the contraction circle, and the definition of $\overrightarrow {A}$, $\overrightarrow {C}$ are as follows^17,26^:

12 $$\left\{ {\begin{array}{*{20}l} {\overrightarrow {A} = 2\overrightarrow {a} \cdot \overrightarrow {r}_{1} - \overrightarrow {a} } \hfill \\ {\overrightarrow {C} = 2 \cdot \overrightarrow {r}_{2} } \hfill \\ {\overrightarrow {a} = 2 - \frac{2t}{{T_{\max } }}} \hfill \\ \end{array} } \right.$$

When WOA set to $A \ge {1}$, the whale is forced to deviate from the prey, so as to find a more suitable prey, which can strengthen the exploration ability of the algorithm and make WOA to conduct global search. The mathematical model is as follows^26^:

13 $$\overrightarrow {X} (t + 1) = \overrightarrow {X}_{rand} - \overrightarrow {A \cdot } \left| {\overrightarrow {C} \overrightarrow {X}_{rand} - \overrightarrow {X} (t)} \right|$$

where $\overrightarrow {X}_{rand}$ is a random selection of whale locations (not a pre-optimal solution).

In GWO_BP, we will use Eqs. (11–13) to replace the position update formula of $\alpha$ wolf.

#### *Lévy flight for the head wolf *$\alpha$

In order to improve the population diversity and avoid falling into the local optimal solution, we use Lévy flight to improve the alpha wolf search strategy for global detection.

Lévy flight^27^ is a random search method that obeys Lévy distribution and Lévy flight is named after Paul Pierre Lévy, the French mathematician. The search step of Lévy flight is a short-distance and long-distance search alternately. Such a search method has good global search capabilities^28^.

The position update equation of Lévy flight is as follows^27^:

14 $$x_{i} (t + 1) = x_{i} (t) + l \oplus Levy(\overline{\lambda })$$

where, $\oplus$ represents point-to-point multiplication, and *l* > 0 is the step size parameter related to the scope of the optimization problem.

As the equation of Lévy flight is very complex, we use Mantegna algorithm to simulate^29,30^. The calculation equation of step size is as follows:

15 $$s_{p} = \frac{\mu }{{\left| \nu \right|^{1/\chi } }}$$

In GWO, the value of α wolf is the closest to the optimal solution, so we add α to $s_{p}$, a new step size formula is obtained, it is a complex process. The step length calculation formula is as follows:

16 $$s = 0.01 \cdot s_{p} \cdot (X_{p} (t) - X_{\alpha } (t))$$

where, $\mu$ and $\nu$ Obey normal distribution, $\mu \sim N(0,\sigma_{\mu }^{2} )$, $\nu \sim N(0,\sigma_{\nu }^{2} )$, $\sigma_{\nu } = 1$, The equation of $\sigma_{\mu }$ is as follows^31^:

17 $$\sigma_{\mu } = \left\{ {\frac{\Gamma (1 + \chi )\sin (\pi \chi /2)}{{\chi \cdot \Gamma \left[ {(1 + \chi )/2} \right] \cdot 2^{(\chi - 1)/2} }}} \right\}^{1/\chi }$$

where the value of $\chi$ is usually 1.5.

#### The GWO_BP

**Figure 1:**
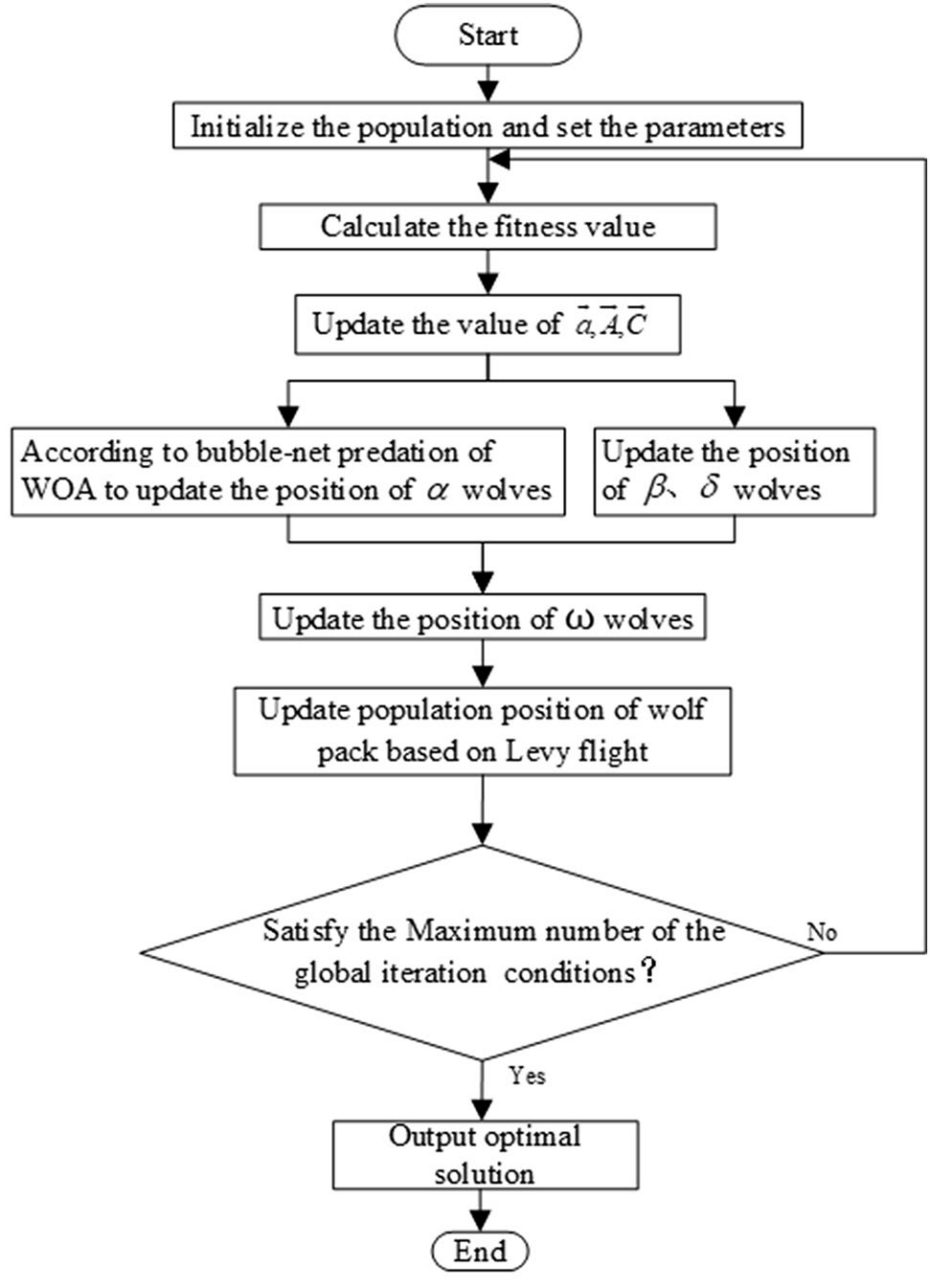
Flow chart of GWO_BP.

From the discussed as above, the flowchart of GWO_BP is shown in Fig. 1, and the procedure of GWO_BP are as follows:

*Step 1* Set the control parameters of the algorithm. They are the population size $S$, the maximum number of iterations $T_{\max }$, the dimension of variables to be optimized $\dim$, the sum of upper $ub$ and lower bounds $lb$ in space, and initial population randomly.

*Step 2* Calculate the fitness of all individuals in the population, sort by fitness, that is, $\alpha$ = the individual with the best fitness, $\beta$ = the individual with the second fitness, $\delta$ = the individual with the third fitness ranking.

*Step 3* Calculate the convergence factor $\overrightarrow {a}$ according to Eq. (5). Calculate $\overrightarrow {A}$, $\overrightarrow {C}$ according to Eqs. (3) and (4). the wolf $\alpha$ has the best fitness value, so in order to improve the convergence speed and global search ability, this paper replaces the position update Eqs. (6–8) of the leading wolf with the whale spiral bubble predation Eqs. (11–13). $\beta ,\delta ,\omega$ wolves update the position according to the original GWO Eqs. (6–8).

*Step 4* Update the position of the wolf pack according to the Lévy flight equation of the head wolf $\alpha$ position in Eqs. (14–17).

*Step 5* Update $$\overrightarrow {a}$$, $$\overrightarrow {A}$$, $$\overrightarrow {C}$$.

*Step 6* Determine whether the number of iterations reaches the maximum $T_{\max }$ or satisfy other algorithm termination conditions. If satisfied, output the current optimal solution, otherwise, return to step 2 and continue.

## Experimental results

### Test function optimization

In order to verify the effectiveness of GWO_BP, twelve test functions in Tables 1, 2 and 3 are selected for the numerical optimization. $f_{1} - \, f_{{4}}$ in Table 1 are unimodal functions, they only have one extreme point, which are mainly used to test the accuracy of the local search and the development ability of the algorithm; $f_{5} - f_{8}$ are multimodal functions, the feature of these functions are that they have multiple local minima, so they also suitable for detecting the global search ability of the algorithm, that is, the detectability of the GWO_BP: whether it has the ability to jump out of the local optimum and search for the global optimum; $f_{9} - f_{12}$ are fixed-dimension multimodal functions, they are also the multimodal functions but the dimension of these functions are fixed.

**Table 1:** Unimodal benchmark functions.

Function name	Function	Bounds	Dim	$F_{\min }$
Sphere	$f_{1} ({\varvec{x}}) = \sum\nolimits_{i = 1}^{n} {x_{i}^{2} }$	$[ - 100,100]$	30	0
Schwefel 2.22	$f_{2} ({\varvec{x}}) = \sum\nolimits_{i = 1}^{n} {\left| {x_{i} } \right|} + \prod\nolimits_{i = 1}^{n} {x_{i} }$	$[ - 10,10]$	30	0
Schwefel 1.2	$f_{3} ({\varvec{x}}) = \sum\nolimits_{i = 1}^{n} {(\sum\nolimits_{j = 1}^{i} {x_{j} } )}^{2}$	$[ - 100,100]$	30	0
Schwefel 2.21	$f_{4} ({\varvec{x}}) = \max_{i} \left\{ {\left| {x_{i} } \right|,1 \le i \le n} \right\}$	$[ - 100,100]$	30	0

**Table 2:** Multimodal benchmark functions.

Function name	Test functions	Bounds	Dim	$F_{\min }$
Schwefel	$f_{5} ({\varvec{x}}) = \sum\nolimits_{i = 1}^{n} { - x_{i} } \sin (\sqrt {\left| {x_{i} } \right|} )$	$[ - 500,500]$	30	− 418.9829*n
Rastrigin	$f_{6} ({\varvec{x}}) = \sum\nolimits_{i = 1}^{n} {\left[ {x_{i} - 10\cos (2\pi x_{i} ) + 10} \right]}$	$[ - 5.12,5.12]$	30	0
Ackley	$f_{7} ({\varvec{x}}) = \sum\nolimits_{(i = 1)}^{n} { - 20exp} ( - 0.2\sqrt {\frac{1}{n}\sum\nolimits_{i = 1}^{n} {x_{i}^{2} } } ) - exp\left( {\frac{1}{n} \, \sum\nolimits_{i = 1}^{n} {cos} \left( {{2}\pi x_{i} } \right)} \right) + 20 + e$	$[ - 32,32]$	30	0
Griewank	$f_{8} ({\varvec{x}}) = \frac{1}{4000}\sum\nolimits_{i = 1}^{n} {x_{i}^{2} - \prod\nolimits_{i = 1}^{n} {\cos (\frac{{x_{i} }}{\sqrt i })} } + 1$	$[ - 600,600]$	30	0

**Table 3:** Fixed-dimension multimodal benchmark functions.

Function name	Test functions	Bounds	Dim	$F_{\min }$
Kowalik	$f_{9} ({\varvec{x}}) = \sum\nolimits_{i = 1}^{11} {\left[ {a_{i} - \frac{{x_{1} (b_{i}^{2} + b_{i} x_{2} )}}{{b_{i}^{2} + b_{i} x_{3} + x_{4} }}} \right]}^{2}$	$[ - 5,5]$	4	0.00030
Hartman 3	$f_{10} ({\varvec{x}}) = - \sum\nolimits_{i = 1}^{4} {c_{i} \exp ( - \sum\nolimits_{j = 1}^{3} {a_{ij} } (x_{j} - p_{ij} )^{2} )}$	$[1,3]$	3	− 3.86
Shekel 7	$f_{11} ({\varvec{x}}) = - \sum\nolimits_{i = 1}^{7} {[(X - a_{i} )(X - a_{i} )^{T} + c_{i} ]^{ - 1} }$	$[0,10]$	4	− 10.4028
Shekel 10	$f_{12} ({\varvec{x}}) = - \sum\nolimits_{i = 1}^{10} {[(X - a_{i} )(X - a_{i} )^{T} + c_{i} ]^{ - 1} }$	$[0,10]$	4	− 10.5363

### Parameter setting

We compare the GWO_BP with the basic GWO, PSO and the basic GWO with Lévy flight (LGWO). The parameter values ​​of the four optimization algorithms are shown in Table 4.

**Table 4:** Parameter setting.

GWO_PB	GWO	PSO	LGWO
$S = 30$	$S = 30$	$S = 30$	$S = 30$
$T_{\max } = 500$	$T_{\max } = 500$	$T_{\max } = 500$	$T_{\max } = 500$
$\alpha_{1} = 1$		$\omega_{\max } = 0.2$	$\alpha_{1} = 1$
$\beta_{1} = 1.5$		$\omega_{\min } = 0.9$	$\beta_{1} = 1.5$
$p_{i} = 0.5$		$V = 6$	
$b = 1$		$c = 2$	

Each algorithm of GWO_BP, GWO and PSO is run 30 times for the 12 test functions to calculate the optimal value, mean value and variance. The results of the three algorithms are shown in Table 5, and the optimal values of the three algorithms are marked in bold.

**Table 5:** The optimization results of GWO_BP, GWO and PSO.

Test function	GWO_BP			GWO			PSO		
	$F_{b}$	$\overline{F}$	$F_{V}$	$F_{b}$	$\overline{F}$	$F_{V}$	$F_{b}$	$\overline{F}$	$F_{V}$
$f_{1}$	**3.39e**−**272**	1.34e−201	0	3.54e−31	1.89e−27	5.19e−27	3.19e−6	0.0010256	0.0002591
$f_{2}$	**1.41e**−**130**	2.63e−105	1.44e−104	1.16e−17	8.33e−17	5.15e−17	0.0033293	0.025077	0.025181
$f_{3}$	**1.47e**−**235**	1.04e−175	0	4.29e−8	6.11e−6	8.83e−6	22.5641	76.8104	29.6565
$f_{4}$	**5.94e**−**125**	7.21e−95	3.94e−94	6.49e−8	7.79e−7	9.31e−7	0.73877	1.1871	0.27429
$f_{5}$	− **11,029.776**	− 11,116.73	5.75e−8	− 6573.7406	− 5906.67	0.41465	− 7792.144	− 5401.569	1395.7081
$f_{6}$	**0**	0	0	**0**	11.8383	3.5676	33.852	55.271	12.4957
$f_{7}$	**8.88e**−**16**	8.88e−16	0	7.55e−14	1.037e−13	1575e−14	0.0033485	0.29308	0.57525
$f_{8}$	**0**	0	0	**0**	0.0042187	0.0086354	1.4178e−6	0.0092825	0.008039
$f_{9}$	**0.00030644**	0.0017249	0.005068	0.0003075	0.003824	0.0075286	0.00050106	0.0008804	0.0001414
$f_{10}$	− **3.8628**	− 3.8628	2.19e−15	− **3.8628**	− 3.8628	0.001398	**− 3.8628**	− 3.8628	2.65e−15
$f_{11}$	− **5.0832**	− 5.0604	0.019028	− 10.4028	− 10.4012	0.0007519	− 10.4029	− 8.1837	3.0366
$f_{12}$	− **10.5364**	− 10.5364	3.5134	− 10.536	− 10.2039	1.4811	**− 10.5364**	− 9.1866	2.569

Significant values are in [bold].

### Results and discussion

From Table 5, for the comparison of the optimal values of unimodal function $f_{1} - \, f_{{4}}$, it can be seen that the optimization accuracy of GWO_BP is far beyond several orders of magnitude of GWO and PSO. Therefore, it can be concluded that the local exploration ability of GWO_BP proposed in this work is much higher performance than that of GWO and PSO. At the same time, comparing the mean value and the variance of the three algorithms, the result of GWO_BP is still far better than GWO and PSO. It also can be concluded that GWO_BP is better than GWO and PSO, which has very strong local search ability and good stability; According to the comparison of the optimal values of multimodal function $f_{5} - f_{8}$ in Table 5, the exploration performance of GWO_BP is better than GWO and PSO. Optimization of GWO_BP for $f_{6}$ and $f_{8}$ has reached the optimal value of the function, and the average results have reached the optimal value of 0, which proves that the difference between most optimization results and their mean results is small, and the optimization results are relatively stable each time. To sum up, it can be concluded that GWO_BP has strong global search ability and stability; Through the optimization of the function with multi peak fixed dimension $f_{9} - f_{12}$, it can be seen from Table 5 that GWO_BP is slightly better than GWO and PSO in the comparison of the optimal value results of the functions.

### Modeling of FCCU main fractionator

#### Description of FCC unit main fractionator

With the gradual development of national economy, heavy oil catalytic cracking has become a very important problem in today's industrial production^32,33^. The FCC fractionation system is mainly composed of the main fractionator, the overhead oil and gas condensation cooling system, the diesel stripper, the recycle tank and its interrupted reflux. In the typical split flow system process, the high-temperature reaction oil vapor mixture at 450–510 °C comes out from the top of the reactor, entrains a small amount of catalyst powder, enters the desuperheating section of the lower section of the main fractionator, contacts with the 250 °C oil slurry countercurrent on the baffle for heat exchange, desuperheates and washes the entrained catalyst powder, and then enters the main body of the fractionator. In the main fractionator, the oil vapor mixture condensed to the saturated state is separated.

#### Process modeling

We apply GWO_BP to the parameter estimation of the FCCU main fractionator model^34^. In the main fractionator of a 1.4 million tonnes heavy oil catalytic cracking unit in a refinery, the reaction oil vapor enters the fractionator from the bottom and is cooled and washed from bottom to top. In order to provide sufficient internal reflux, remove the heat in the tower and make the load distribution of the tower uniform, the fractionator is equipped with four thermal cycle systems, namely, top exhaust heat cycle, first middle exhaust heat cycle, second middle exhaust heat cycle and slurry exhaust heat cycle. The factors that affect the dry point are the top temperature, top pressure, top heat discharge and other related parameters, while the factors that affect the pour point are the top load change, the top pressure change, the first and second heat discharge. The change of heat discharge is realized by the change of flow or the change of temperature, so we set the controlled variables are the top temperature *y*_1_, the crude gasoline dry point *y*_2_, the pour point of light diesel oil *y*_3_; the control variables are the circulating flow *u*_1_, the first medium flow *u*_2_, the second middle flow *u*_3_. 100 input and output data are used for the parameter modeling; The dynamic model of FCCU main fractionation column process is as follows^34^:

18 $$\begin{aligned} y_{1} (z^{ - 1} ) & = \frac{{a_{11} (1) + a_{11} (2)z^{ - 1} }}{{1 + b_{11} z^{ - 1} }}z^{{ - d_{11} }} u_{1} (z^{ - 1} ) \\ & \quad + \frac{{a_{21} (1) + a_{21} (2)z^{ - 1} }}{{1 + b_{21} z^{ - 1} }}z^{{ - d_{21} }} u_{2} (z^{ - 1} ) \\ \end{aligned}$$

19 $$\begin{aligned} y_{2} (z^{ - 1} ) & = \frac{{a_{22} (1) + a_{22} (2)z^{ - 1} }}{{1 + b_{22} z^{ - 1} }}z^{{ - d_{22} }} u_{2} (z^{ - 1} ) \\ & \quad + \frac{{a_{32} (1) + a_{32} (2)z^{ - 1} }}{{1 + b_{32} z^{ - 1} }}z^{{ - d_{32} }} u_{3} (z^{ - 1} ) \\ \end{aligned}$$

20 $$y_{3} (z^{ - 1} ) = \frac{{a_{33} (1) + a_{33} (2)z^{ - 1} }}{{1 + b_{33} z^{ - 1} }}z^{{ - d_{33} }} u_{3} (z^{ - 1} )$$

Due to the coupling between $y_{1}$ and $y_{{2}}$, Zhong et al. established the model of the main fractionation column of the FCCU based on the field data of typical FCC system. Therefore, parameters of FCCU dynamic mathematical model provided by Zhong et al. are used^34^.

**Figure 2:**
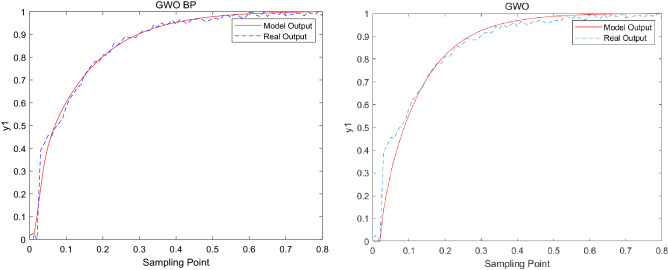
Modeling result graph of *y*_*1*_*.*

**Figure 3:**
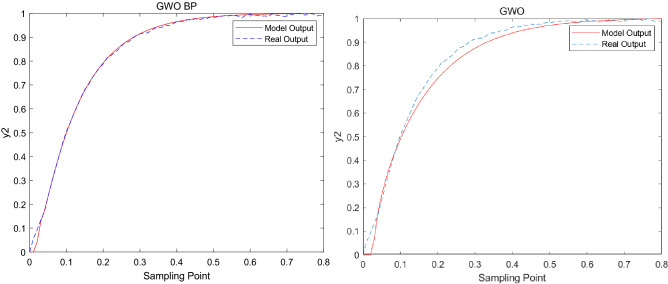
Modeling result graph of *y*_2_.

**Figure 4:**
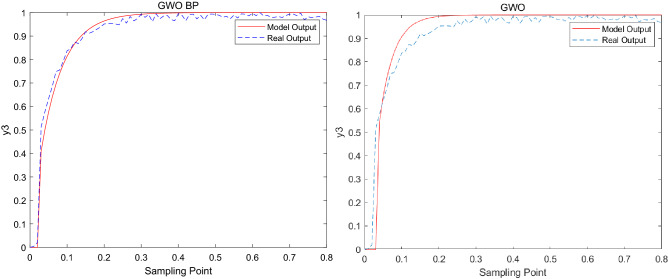
Modeling result graph of *y*_3_.

GWO_BP, LGWO and GWO are used to estimate the model parameters in the main fractionator and the algorithms parameter setting is shown in Table 4. The modeling results are shown in Figs. 2, 3 and 4.

The model error formula is as follows^35^:

21 $$e_{{_{i} }} = \frac{1}{N}\sum\limits_{k = 1}^{N} {\left| {y_{i} (k) - \hat{y}_{i} (k)} \right|} ,\begin{array}{*{20}c} {} & {i = 1,2,3} \\ \end{array}$$

where $N$ is the number of samples, $\hat{y}_{i}$ is the model output, $y_{i}$ is the model actual output. The adjustable range of $y_{1}$ ~ $y_{3}$ is 0.0–0.8, and the allowable error range is within 0.1. The output error of GWO_BP, LGWO and GWO are shown in the Table 6.

**Table 6:** The output error.

Methods	$e_{1}$	$e_{2}$	$e_{3}$
GWO_BP	0.01292	0.00487	0.01009
LGWO	0.01443	0.00593	0.04754
GWO	0.03548	0.01629	0.07486

From the modeling results in Figs. 2, 3 and 4, it can be seen that the modeling results of this method reflect the actual characteristics of the actual system well, while the modeling results of GWO are far less ideal than that of GWO_BP. From the error analyzed in Table 6, it can be seen that the error of the improved algorithm is closer to 0. In addition, in order to further verify the effectiveness of the new algorithm in model building, the error is compared with LGWO. The experimental results show that the output error of GWO_BP is less than LGWO.

## Conclusions

To realize the advanced control of product quality in a refinery is to establish a high precision dynamic model of complex industrial process. In this work, by combining bubble-net predation of whales and Lévy flight, a novel GWO (GWO_BP) is proposed for parameter estimation of FCCU main fractionator model. The new algorithm can make up for the imbalance between exploration and development of the original GWO. On the one hand, the whale bubble predation method is replaced by the surrounding predation method of the head wolf $\alpha$ in GWO to enhance the global search ability; On the other hand, the Lévy flight improved the head wolf $\alpha$ search strategy is used to iteratively update the wolf swarm to overcome the disadvantage of the algorithm falling into local optimization, so as to speed up the convergence speed and improve the convergence accuracy of the algorithm as a whole. GWO_BP, GWO and PSO are applied to 12 typical test functions. The experimental results show that the performance of GWO_BP is much better than the others. And compared with basic GWO and LGWO, the results reveal that the FCCU main fractionator model predictive outputs of GWO_BP are in better agreement with the actual experimental data.
